# Drug-based pain management for people with dementia after hip or pelvic fractures: a systematic review

**DOI:** 10.1186/s12877-017-0446-z

**Published:** 2017-02-14

**Authors:** Kai Moschinski, Silke Kuske, Silke Andrich, Astrid Stephan, Irmela Gnass, Erika Sirsch, Andrea Icks

**Affiliations:** 1 Heinrich Heine University, Faculty of Medicine, Institute for Health Services Research and Health Economics, Moorenstraße 5, 40225 Düsseldorf, Germany; 2 Faculty of Nursing Science, Vallendar College of Philosophy and Theology (PTHV Catholic University), Palottistraße 3, 56179 Vallendar, Germany; 3 Department of Nursing, University Hospital Aachen, Pauwelsstraße 30, 52074 Aachen, Germany

**Keywords:** Pain management, Analgesics, Drugs, Hip fractures, Pelvic fractures, Dementia, Alzheimer, Cognitive impairment, Cognitive disorders

## Abstract

**Background:**

Studies indicate that people with dementia do not receive the same amount of analgesia after a hip or pelvic fracture compared to those without cognitive impairment. However, there is no systematic review that shows to what extent drug-based pain management is performed for people with dementia following a hip or pelvic fracture.

The aim of this systematic review was to identify and analyse studies that investigate drug-based pain management for people with dementia with a hip or pelvic fracture in all settings. Treatment could be surgical or conservative. We also analysed study designs, methods and variables, as well as which assessments were applied to measure pain management and mental status.

**Method/design:**

The development of this systematic review protocol was guided by the PRISMA-P requirements, which were taken into consideration during the review procedures. MEDLINE, EMBASE, CINAHL, Web of Knowledge and ScienceDirect were searched. Studies published up to January 2016 were included. The data extraction, content and quantitative descriptive analysis were carried out systematically, followed by a critical appraisal.

**Results:**

Eight of the 13 included studies focusing on patient data showed that people with dementia received less drug-based pain management than people without cognitive impairment. Four studies based on surveys of healthcare professionals stated that cognitive impairment is a major barrier for effective pain management. There was heterogeneity regarding the assessment of the mental status and the pain assessment of the patients. The assessment of the drugs administered in all of the studies working with patient data was achieved through chart reviews.

**Conclusion:**

People with dementia do not seem to receive the same amount of opioid analgesics after hip fracture as people without cognitive impairment. There is need to enhance pain assessment and management for these patients. Future research should pay more attention to the use of the appropriate items for assessing cognitive impairment and pain in people with dementia.

**Trial registration:**

This systematic review was registered at Prospero (CRD42016037309); on 11 April 2016, and the systematic review protocol was published (Syst Rev. 5(1):1, 2016).

## Background

The burden and relevance of osteoporotic fractures are expected to increase due to worldwide demographic changes and an ageing population [1]. Hip and pelvic fractures are often a consequence of falls [2–4] and represent around 13% of all fractures [5]. Fall-related fractures among older people seem to be frequently associated with dementia [6] and the risk of suffering one of the two fractures rises significantly as from the age of 65 years [5].

The number of people with dementia (PwD) is increasing significantly [7], comparable to the incidence of hip and pelvic fractures, beginning at the age of 60 [8]. PwD have a two to three times higher risk of a fall-related fracture than cognitively intact people, which is due to sensory or muscular restrictions [9].

Pain management after a fracture is crucial for PwD [10]. A study by Husebo et al. [11] examining 352 nursing home residents with moderate to severe dementia concluded that a standardised stepwise protocol of treatment with analgesics for these patients significantly improved not only pain, but also neuropsychiatric symptoms and agitation. Morrison and Siu [12] showed that the majority of the participants diagnosed with dementia who had previously experienced a hip fracture received only one-third of the analgesic drugs that are given to cognitively intact patients. Studies have pointed out that PwD, compared with those without dementia, are often not able to verbalise the pain [13, 14], and have an increased sensitivity to pain at the same time [15]. Furthermore, persistent pain can lead to functional decline, social isolation, depression, increased healthcare utilisation and delirium [13]. However, there are no systematic reviews showing to what extent drug-based pain management is performed for people with dementia following a hip or pelvic fracture.

## Objectives

The aim of this systematic review was to identify and analyse studies that investigate drug-based pain management for people with dementia with a hip or pelvic fracture in all settings. Treatment could be surgical or conservative. We also analysed study designs, methods and variables, as well as which assessments were applied to measure pain management and mental status.

## Method/design

This systematic review was performed in line with the quality requirements of the PRISMA-P guideline. It was registered at Prospero (CRD42016037309), and the systematic review protocol, including details and the complete search algorithm, has been published [16].

### Search strategy

The search was performed according to the protocol, and studies were included that investigated drug-based pain management for PwD following hip or pelvic fractures in all settings and which were published up to January 2016. MEDLINE, EMBASE, CINAHL, Web of Knowledge and ScienceDirect were searched. The search strategy was set up using the database-specific vocabularies (MeSH, EMTREE), additional free text terms and Boolean operators (AND, OR). Included search terms were, for example, “analgesia”, “dementia”, “cognitive impairment”, “pain treatment”, “hip fracture” or “pelvic fracture”. Study selection was performed independently by two reviewers who screened the titles and abstracts. The selected full texts were also double-checked for inclusion. Backward citation tracking and forward citation tracking were performed. The complete search strategy can be found in the systematic review protocol [16].

### Inclusion and exclusion criteria

Titles and abstracts had to be in English or German. Studies were included that investigated drug-based pain management for people with dementia in cases of hip or pelvic fracture which were treated either by operation or conservatively, regardless of the setting. Original qualitative, quantitative or mixed-methods studies and also grey literature were included. Letters, short reports and abstracts were screened in order to identify further original studies. Publications without available references were excluded. The inclusion criteria were pre-tested with a set of 100 articles. Studies with a high level of bias were finally excluded after critical appraisal.

### Data analysis

Data was extracted to gain an overview of the studies’ contents, i.e., study design, settings and study findings. Further, we extracted data collection methods, outcomes measures, type of data, e.g. databases on administrative or clinical data etc., regarding the applied assessments of mental status, pain and drugs in the identified studies. A content analysis was conducted deductively with predefined categories: e.g. author, date, study type, study designs, mental tests, pain scales, findings and the result of the critical appraisal of the study quality. In order to describe the pain management and the applied assessments, further categories were developed inductively by identifying the relevant aspects of the studies, e.g. categorisation of drugs in the studies, assessment of the administered drugs.

### Quality assessment

A critical appraisal was performed by two reviewers independently for each type of study by using specific appraisal tools and the checklists of the Scottish Intercollegiate Guideline Networks (SIGN) [17]. Studies that were not covered by the SIGN guidelines were evaluated, using the tools of the National Institute for Health and Care Excellence (NICE) for cross-sectional studies [18]; tools from the Joanna Briggs Institute for Case Series [19] and tools developed by Greenhalgh et al. [20] were applied for questionnaire surveys.

## Results

**Fig. 1:**
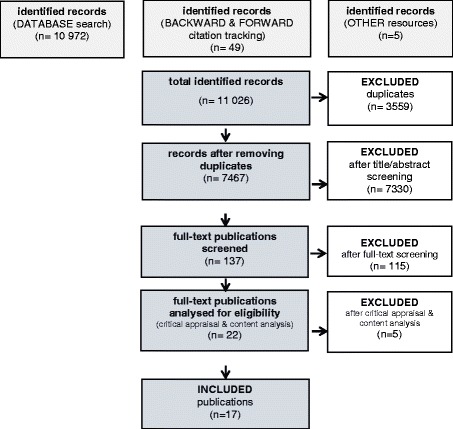
Flow chart

A total of 7467 records were identified, out of which 17 studies were included that addressed drug-based pain management for PwD after hip fracture, but none for the same topic following pelvic fractures (see Fig. 1). The interrater reliability was 99,34% for title and abstract screening. Differences were resolved. The two raters completely agreed regarding the full text selection. An overview of the included studies can be found in Table 1.

**Table 1:** Overview of the included studies (for methods and data analysis see Table 3)

Author, year	Setting/country	Objectives	Findings
COHORT STUDIES			
Adunsky et al. 2002 [10]	hospital/Israel	Are PwD treated differently to those without cognitive impairment, and what factors might affect this?	PwD received only 53% of the amount of opioid that was administered to cognitively intact patients. Significant association between cognitive status and amount of opioid analgesia.
Feldt et al. 1998 [33]	hospital/USA	Experience and treatment of pain in PwD vs. those without cognitive impairment?	Prescription of pain medication did not differ significantly, but cognitively impaired subjects received fewer opioid analgesics. Both groups received less than 25% of the mean prescribed amount of opioid analgesics.
Feldt et al. 2000 [35]	hospital/USA	Is post-operative pain a predictor of functional outcomes for elderly hip fracture patients who were previously independent ambulators?	Undertreated post-operative pain contributes to poor functional outcomes. No differences between PwD and those without cognitive impairment in the amounts of opioid analgesics or acetaminophen prescribed or administered in the first or second 48 h post-op.
Feldt & Gunderson 2002 [29]	across settings/USA	Observing the treatment of pain following hip fracture across settings.	Subjects received significantly less medication during the first 24 h in the nursing home (NH) as compared with the last 24 h in the hospital. Over one-thrid of the subjects received no opioid analgesics and 18.3% received no analgesic of any kind during the first 24 h of NH stay. 91.5% of the opioid analgesics were prescribed PRN. Subjects in the hospital setting received more analgesia regardless of MMSE score. Setting is the only relevant factor.
Grall 2010 [34]	hospital/USA	Are there differences in pain expression, assessment and management in hospitalised elderly persons?	Pain in PwD is under-recognised and undertreated in the acute care setting, and current clinical practice guidelines with regards to pain assessment are not being followed. People without dementia received almost 50% more pain medication compared with their counterparts with dementia following acute hip fracture.
Jensen-Dahm et al. 2016 [22]	hospital/Denmark	Do hip fracture patients with dementia receive less post-operative pain treatment than those without cognitive impairment?	PwD received lower doses of oral morphine equivalents during the first and second post-operative day, lower doses of acetaminophen during the first 3 days post-op, and were more likely to receive opioids PRN.
McDermott et al. 2014 [26]	ED/UK	To identify inconsistencies in pain management within the acute setting.	PwD received a weaker level of analgesia both in the ambulance and in the accident and emergency setting.
Morrison & Siu 2000 [12]	hospital/USA	Observation of the treatment of pain following hip fracture.	Advanced dementia patients received one-third of the amount of morphine sulphate equivalents received by the cognitively intact patients. 76% of the PwD and 83% of the cognitively intact patients did not have a standing order for their analgesic agent during their entire hospitalisation.
CROSS-SECTIONAL STUDIES			
Holdgate et al. 2010 [27]	ED/Australia	To identify patterns of analgesia administered and real or potential barriers to providing analgesia after hip fracture.	Cognitive impairment and language difficulties as most reported barriers.
Hwang et al. 2006 [28]	ED/USA	What is the effect of emergency-department crowding on assessment and treatment of pain in older adults?	Dementia as a risk factor for undertreatment of pain, considerable delays in analgesic administration, and treatment with inappropriate analgesics.
Mak et al. 2011 [31]	hospital/Australia	Observation of analgesia use among patients with hip fracture requiring surgery in correlation to hip fracture subtype, cognitive status and type of surgery in the post-acute period.	PwD utilised markedly less analgesia at all time periods measured.
Titler et al. 2003 [32]	hospital/USA	Observation of acute pain management practices for patients hospitalised for hip fracture.	Only 27% received patient-controlled analgesia and only 22.3% received around-the-clock administration during the first 24 h after admission of analgesics that had been ordered PRN. PwD received significantly less mean parental morphine equivalents of opioids than those without dementia.
CASE SERIES			
Ardery et al. 2003 [21]	hospital/USA	Why did eight patients recruited from a previous study (Titler et al. 2003 [32]) receive no opioid during the first 72 h after admission?	Mental status cannot by itself account for patterns of analgesic administration.
HEALTHCARE PROFESSIONALS’ SURVEYS			
Rantala & Kankkunen et al. 2012 [14]	hospital/Finland	Common aim of both studies: to identify current post-operative pain management practices for PwD and hip fracture; barriers to post-operative pain management in hip fracture PwD; nurses’ expectations and facilitators offered by employers to overcome barriers in pain management.	The major barrier to effective pain management was stated to be difficulties in assessing pain because of a decline in cognition.
Rantala & Kankkunen et al. 2014 [23]			
Rantala & Hartikainen et al. 2014 [25]	hospital/Finland	Common aim of both studies: to identify the analgesic use in hip fracture PwD during the first two post-operative days as reported by nurses, and nurses’ knowledge regarding relevant adverse effects of different types of analgesics when treating post-operative pain in PwD.	Nurses older than 50 and with over 15 years of work experience in healthcare had complete pain relief as the main goal of pain management significantly more often than younger nurses with less work experience in healthcare.
Rantala & Hartikainen et al. 2015 [24]			

Thirteen out of the 17 included studies focused on patient data. These studies included a total of 4249 patients, of whom 75% were female; 25% of all the participants were cognitively impaired with a sample range between eight [21] to 1507 participants [22]. The mean age of the people with cognitive impairment was 82 years. Only patients aged 52 years or older were included. The participants had various comorbidities and all kinds of hip fractures, and were treated with an operative intervention as well as conservatively. An extraction sheet with the characteristics of the patients can be found in the Table 2. The four studies by Rantala [14, 23–25] were not listed in this table since they focused only on healthcare professionals.

**Table 2:** Characteristics of the participants (only patients)

Author, year	Fracture type	Treatment		Dementia type	Comorbidities	Age		Female
		conser-vative	surgery			mean	range	(%)
COHORT STUDIES								
Adunsky et al. 2002 [10]	hip fractures: 112 trochanteric, 72 subcapital	-	✔	38 delirium 50 dementia	-	81	63–100	74
Feldt et al. 1998 [33]	-	-	✔	not reported	comorbidities: 3.1 (mean) Illness severity score: 5 (mean)	86	≧65	86
Feldt et al. 2000 [35]		-	✔	not reported	comorbidities: 3.5 (mean) Illness severity score: 5.1 (mean)	84	≧65	91
Feldt & Gunderson 2002 [29]		-	✔	not reported	comorbidities: 3.3 (mean) Illness severity score: 5 (mean)	85	≧65	88
Grall 2010 [34]	-	-	-	49 Alzheimer dementia 23 non-specified dementia	120 cardiovascular 32 cerebrovascular 87 endocrine 57 musculoskeletal 47 neuro non-vase 29 pulmonary sum: 3.3	86	65–85	86
Jensen-Dahm et al. 2016 [22]	1025 displaced 737 femoral neck	-	✔	not reported	ASA score: I (136), II (877), ≥ III (494) 286 osteoporosis 395 cancer 221 contralateral hip fracture	83	≥65	74
McDermott et al. 2014 [26]	femoral neck	✔	-	not reported	-	82	-	84
Morrison & Siu 2000 [12]	hip fractures: 54 femoral neck, 41 intertrochanteric	✔	✔	98 severe and very severe (Reisberg scale 6 and 7)	excluded if concomitant diagnosis of cancer, multiple internal injuries or previous fracture in the affected hip	83	71–100	81
CROSS-SECTIONAL STUDIES								
Holdgate et al. 2010 [27]	femoral neck	-	-	not reported	21 patients had documented comorbidities	76	-	68
Hwang et al 2006 [28]	hip fractures	-	-	not reported	RAND comorbidity score, mean +/- SD (range 0–12) 2.7 +/- 2.2	83	52–101	80
Mak et al. 2011 [31]	220 trochanteric (136 stable, 84 unstable), 195 subcapital (39 undisplaced; 156 displaced)	-	✔	54 mild 48 moderate 52 severe	37 previous hip fracture 158 previous documented osteoporosis or an atraumatic fracture	81	60–100	74
Titler et al. 2003 [32]	-	-	-	158 Alzheimer dementia	150 atherosclerosis 102 heart failure 62 ulcer 61 alcohol use 44 renal impairment 35 gastrointestinal bleeding 15 liver impairment 5 increase intracranial pressure 2 abnormal platelet function	83	65–103	77
CASE SERIES								
Ardery et al. 2003 [21]	5 pertrochanteric, closed, 1 transcervical, closed 1 femoral neck, closed 1 femoral neck, open	-	✔	not reported	2 renal disease 2 ulcer disease 4 arteriosclerotics 2 consecutive heart failure 1 alcohol use	80	67–92	62.5

All but four of the studies were performed in a hospital setting: McDermott et al. [26], Holdgate et al. [27], and Hwang et al. [28] were performed in an emergency department setting; Feldt and Gunderson [29] performed their study across settings (hospital to nursing home or rehabilitation facility).

### Critical appraisal results

Out of the 22 full texts selected, five with considerable methodological flaws were excluded: for example, if the inclusion criteria were unclear [13], or if no confounders were considered and the outcomes were not clearly defined [30]. The eight included cohort studies with a lower number of bias met six to nine of the 14 quality criteria of the SIGN checklist. It is to be taken into account that in the study by Jensen-Dahm et al. [22], which met six quality criteria, five of the items were “not applicable” in terms of the critical appraisal. Three of the four cross-sectional studies met 11 quality criteria [28, 31, 32], while the study by Holdgate et al. [27] met 10 of the 17 quality criteria of the NICE checklist. The case study by Ardery et al. [21] met seven out of the ten quality criteria of the Joanna Briggs Institute’s checklist. Three of the four healthcare professionals’ surveys studies by Rantala met eight of the 13 quality criteria of Greenhalgh’s checklist [14, 24, 25]. The study by Rantala and Hartikainnen [23, 25] met four points since some facts were not reported. However, they were reported in a similar study by the authors a year later [24]. A more detailed look at the critical-appraisal results can be found in Table 3.

**Table 3:** Quality of the included studies

Author, year	Methods	Type of data			Data analysis		Primary outcome	Recruitment/data collection	Partici-pants	Critical appraisal		
		clinical	adminis-trative	inter-view					(PwD)	checklist	“yes”	“not applicable”
COHORT STUDIES												
Adunsky et al., 2002 [10]	retrospective chart review	✔	✔	-	° Pearson correlation test (age/gender/length of stay/type of fracture/dose of administered opiates) ° regression analysis		✔	2 years/1998–2000	184 (50)	SIGN	8/14	5/14
Feldt et al. 1998 [33]	prospective comparative survey	✔	✔	✔	° chi square analysis ° Mann-Whitney test ° Student’s *t*-test ° multivariate regression analysis		✔	9 months/Feb–Oct 1995	88 (53)	SIGN	7/14	1/14
Feldt et al. 2000 [35]	prospective comparative survey	✔	✔	✔			-	9 months/Apr–Dec 1997	85 (40)	SIGN	8/14	1/14
Feldt & Gunderson 2002 [29]	secondary data analysis	✔	-	-			-	18 months/Feb–Oct 1995; Apr–Dec 1997	173 (93)	SIGN	8/14	3/14
Grall 2010 [34]	retrospective chart review	✔	✔	-	° descriptive statistics ° ANOVA-Test ° analysis of variance ° chi square analysis ° regression analysis		✔	5 years/2005–2009	135 (72)	SIGN	7/14	5/14
Jensen-Dahm et al. 2016 [22]	retrospective chart review	-	✔	-			✔	1 year/2009	1507 (296)	SIGN	6/14	5/14
McDermott et al. 2014 [26]	retrospective chart review	✔	-	-	° *t*-test or x^2 test		✔	1 year/June 2011–June 2012	224 (64)	SIGN	7/14	3/14
Morrison & Siu 2000 [12]	prospective chart review + interviews	✔	-	✔	° *t*-test ° chi square analysis ° multivariate regression analysis		✔	18 months/1 Sept 1996–1 Mar 1998	98 (38)	SIGN	9/14	2/14
CROSS-SECTIONAL STUDIES												
Holdgate et al. 2010 [27]	retrospective chart review	✔	✔	-	° means with standard deviations (normally distributed variables) ° medians and interquartile ranges (continuous variables) ° x^2 test (categorical variables) ° Kruskall-Wallis test (time variables not normally distributed)	-		1 year/June 2006–May 2007	646 (42)	NICE	10/17	6/17
Hwang et al. 2006 [28]	retrospective chart review	✔	✔	-	° log transformations ° bivariate Peason correlation test ° t-test ° chi square analysis ° logistic and linear regression models for multivariate analyses	-		1 year/Aug 1997–July 1998	158 (54)	NICE	11/17	5/17
Mak et al. 2011 [31]	prospective chart review	✔	-	-	° chi square analysis ° ANOVA test	✔		1 year/Jan–Dec 2007	415 (154)	NICE	11/17	6/17
Titler et al. 2003 [32]	retrospective chart review	✔	-	✔	° intraclass correlations (continuous variables) ° kappa statistic (categorical data) ° tetrachoric correlation coefficients	✔		1 year/ 1 Jan–31 Dec 1999	709 (185)	NICE	11/17	6/17
CASE SERIES												
Ardery et al. 2003 [21]	retrospective chart review	✔	-	✔	° not applicable		✔	1 year/1 Jan–31 Dec 1999	8 (5)	JBI	7/10	1/10
HEALTHCARE PROFESSIONALS´ SURVEYS												
Rantala & Kankkunen et al. 2012 [14]	descriptive questionaire	-	-	✔	° Likert scale ° *t*-test analysis of variance ° Speaman’s correlation ° qualitative data and content analysis ° explanatory factor analysis via Varimax rotation ° Kaiser-Meyer-Olkin measure ° Bartlett test of sphericity ° four-factor solution		N/A	2 months/March–May 2011	N/A	Green-halgh	8/13	0/13
Rantala & Kankkunen et al. 2014 [23]	descriptive questionaire						N/A				8/13	0/13
Rantala & Hartikainen et al. 2014 [25]	descriptive questionaire	-	-	✔	° chi square analysis ° Mann-Whitney U test ° Kruskal-Wallis test ° qualitative content analysis		N/A	2 months/March–May 2011	N/A	Green-halgh	4/13	0/13
Rantala & Hartikainen et al. 2015 [24]	descriptive questionaire				° logistic regression analysis (Wald Forward method) ° deviation analysis via odds ratio		N/A				8/13	0/13

### Drug-based pain management

Eight of the 13 studies focusing on patient data showed that PwD received less drug-based pain management than people without cognitive impairment. Seven of the eight studies found the differences to be statistically significant, and one study found no significant differences but did note a tendency [33]. These eight studies are as follows: Adunsky et al. [10] stated that PwD received only 53% of the amount of opioid analgesics that were administered to cognitively intact patients (*P* < 0.001); Feldt et al. [33] came to the conclusion that, even though the prescription of pain medication did not differ significantly, PwD received fewer opioid analgesics (*P* = 0.02 in the first and *P* = 0.07 in the second 48 h post-operatively); Grall [34] pointed out that people without dementia received almost 50% more pain medication compared to their counterparts with dementia following acute hip fracture (*p* = 0.018); Jensen-Dahm et al. [22] came to the result that PwD received lower doses of oral morphine equivalents during the first (*P* = 0.001) and second postoperative day (*P* = 0.019), lower doses of acetaminophen during the first 3 days post-operatively (*P* < 0.0001), and were also more likely to receive opioids *pro re nata* (PRN) (*P* = 0.0005); the study by McDermott et al. [26] outlined that PwD received a weaker level of analgesia both in the outpatient and in the emergency setting (*P* < 0.001); Morrison and Siu [12] found that the advanced dementia patients in their study received one-third of the amount of morphine sulphate equivalents that the cognitively intact patients did (*P* < 0.02), and that 76% of the PwD did not have a standing order for their analgesic agent for their entire hospitalisation (*P* = 0.44); in the study by Mak et al. [31]. PwD used distinctly less analgesia during all the time periods measured (*P* < 0.001) and Titler et al. [32] concluded that PwD received significantly fewer mean parenteral morphine equivalents of opioids than those without dementia (*P* < 0.001).

There were five studies that did not identify undertreatment of pain for PwD: Ardery et al. [21] did not find a correlation between cognitive status and patterns of analgesic administration; Feldt et al. [35] found no differences between PwD and those without cognitive impairment in the amounts of opioid analgesics and acetaminophen prescribed or administered in the first or second 48 h post-operatively; and Feldt and Gunderson [29] stated that the setting was the only relevant factor relating to drug-based pain management, arguing that subjects in the hospital setting received more analgesia regardless of their mental status (*P* = 0.025). Despite finding no undertreatment of pain, Hwang et al. [28] still came to the conclusion that dementia is a risk factor for undertreatment of pain, considerable delays in analgesic administration and inappropriate choice of drugs, while Holdgate et al. [27] stated that dementia is one of the most frequently reported barriers to providing analgesia after hip fracture.

The two studies by Rantala and Kankunnen [14, 25] based on surveys of healthcare professionals also concluded that cognitive impairment is a major barrier for effective pain management. Rantala and Hartikainen [14, 25] claimed that the pain medication, if only prescribed PRN, depends heavily on the nurses in charge. More experienced nurses had complete pain relief as the main goal of pain management more often than younger nurses with less work experience [23, 24].

### Assessment of mental status, pain and administered drugs

An overview of clinical assessments and data describing pain management are displayed in Table 4.

**Table 4:** Assessment of mental status, pain and administered drugs

Author, year	Mental tests	Pain scales	Drugs	
			categorisation in the studies	assessment
COHORT STUDIES				
Adunsky et al. 2002 [10]	MMSE; CAM	-	morphine equivalents*1	retrospective survey of opioid consumption during entire hospitalisation
Feldt et al. 1998 [33]	MMSE	FPEI; VDS; CNPI	morphine equivalents*2	retrospective chart review
Feldt et al. 2000 [35]				
Feldt & Gunderson 2002 [29]				
Grall 2010 [34]	ICD-9 codes	verbal: verbal pain intensity (24 h recall period) scores on W-BFPS *5 non-verbal: behavioral pain intensity Scores on the FLACC physiological: changes in HR and SBP	acetaminophen equivalents	retrospective chart review
Jensen-Dahm et al. 2016 [22]	chart review: ICD-8 & -10 codes prescription of antidementia drugs	-	morphine equivalences	retrospective chart review
McDermott et al. 2014 [26]	AMTS after arrival at emergency department	-	WHO analgesic ladder	retrospective chart review
Morrison & Siu 2000 [12]	MMSE; CAM; Reisberg Global Deterioration Scale	VRS (daily assessment)	morphine equivalences WHO analgesic ladder; Pain Management Index (PMI)	daily chart review
CROSS-SECTIONAL STUDIES				
Holdgate et al. 2010 [27]	ICD10-codes	no standardised pain scales (VAS, NRS, Likert scale)	-	retrospective chart review
Hwang et al. 2006 [28]	patient self-reporting of Alzheimer’s disease or other dementia, or physician chart note of dementia	physician’s recording of pain evaluation or the reporting of pain in the patient history or during physical examination	opioid vs. non-opioid (NSAD, acetaminophen)	retrospective chart review
Mak et al. 2011 [31]	MMSE, previously documented dementia;	-	morphine equivalences	retrospective chart review
Titler et al., 2003 [32]	chart review → Medical Record Abstraction Form (MRAF) *3	nurse questionnaire *4	morphine equivalences	retrospective chart review
CASE SERIES				
Ardery et al. 2003 [21]	chart review → MRAF *3	nurse questionnaire *4	morphine equivalences	retrospective chart review
HEALTHCARE PROFESSIONALS´ SURVEYS				
Rantala & Kankkunen et al. 2012 [14]	-	behavioural observation; VAS, VRS, NRS; verbal assessment; facial pain scale; PAINAD	-	
Rantala & Kankkunen et al. 2014 [23]				
Rantala & Hartikainen et al. 2014 [25]	-	-	WHO analgesic ladder; *6	
Rantala & Hartikainen et al. 2014 [25]				

Mental tests: Mini Mental State Examination (*MMSE*); Confusion Assessment Method (*CAM*). Pain tests: Ferrell’s Pain Experience Interview (*FPEI*); Numeric Rating Scale (*NRS*); Visual Analog Scale (*VAS*); Verbal Descriptor Scale (*VDS*); Verbal Rating Scale (*VRS*); Checklist of Non-Verbal Pain Indicators (*CNPI*) — Modified form of the University of Alabama Pain Behaviour Scale; Pain Assessment in Advanced Dementia Scale (PAINAD). Others: Functional status index (*FSI*)

*1 Examined only use of opioids, not simple analgesics such as paracetamol, 10 mg of intramuscular morphine were considered equal to 75 mg of intramuscular meperidine, and to 3 mg of oral morphine sulphate; *2 daily morphine equivalent calculations by Faherty & Grier, since all subjects were given acetaminophen, the daily administered dose was calculated separately; *3 similar to forms used in adherence to AHCPR Acute Pain Guidelines; *4 developed for this study; included a demographic section, the Perceived Stage of Adoption Instrument and the Barriers to Optimal Pain Management Scale; *5 W-BFPS: Wong-Baker FACES Pain Scale (modified by the Bapist Health Care System), *FLACC* Faces, Legs, Activity, Cry, Consolability, *HR* heart rate, *SBP* systolic blood pressure; *6 *DDD* (defined daily doses) by the Anatomical Therapeutic Chemical (*ATC*) Classification System recommended by WHO

A number of different tools were applied to assess the patients’ mental status. The Mini Mental State Examination (MMSE), which was performed most frequently, was used in six studies [10, 26, 29, 31, 33, 35]. Morrison and Siu [12] combined the MMSE with the Confusion Assessment Method (CAM) and the Reisberg Global Deterioration Scale to screen their patients not only for dementia but also for delirium, while Adunsky et al. [10] only combined the MMSE and the CAM.

In the study performed by Mak et al. [31] previously documented dementia and MMSE scores were used to determine the patients’ mental status. McDermott et al. [26] applied the Abbreviated Mental Test Scores (AMTS) to their patients upon arrival at the ambulance and/or emergency department. Further, three studies reviewed the charts for ICD codes for presence of dementia [22, 27, 34], among which the study by Jensen-Dahm et al. [22] also checked for prescription of anti-dementia drugs. Hwang et al. [28] used patients’ self-reports of Alzheimer’s disease or other forms of dementia in combination with physicians’ chart notes regarding mental state, while the study by Titler et al. [32] and its subsequent study by Ardery et al. [21], which worked with the same data, simply reviewed the charts for any sign of dementia. The studies focusing on healthcare professionals did not provide any information about the assessment of their patients’ mental state [14, 23–25].

In four of the included studies focusing on patient data, no information about assessment was given [10, 22, 26, 31]. In the nine studies that actively assessed the patients’ pain there was a great heterogeneity regarding the applied tools. The three studies performed by Feldt et al. [29, 33, 35] applied Ferrel’s Pain Experience Interview (FPEI), as well as the Verbal Descriptor Scale (VDS) and the Checklist of Non-Verbal Pain Indicators (CNPI). The study by Grall [34] assessed pain on three different levels: verbal pain assessment on the Wong-Baker FACES Pain Scale (W-BFPS), non-verbal pain assessment with behavioral pain intensity scores on the Faces, Legs, Activity, Cry and Consolability Scale (FLACC) and physiological changes in heart rate and systolic blood pressure. Morrison and Siu [12] used the Verbal Rating Scale (VRS) daily, while there were four studies that reviewed the charts for all types of pain assessment, because of the heterogeneity of the pain assessments in these hospitals [21, 27, 28, 32]. Two of the studies by Rantala [14, 25], as well as the study by Titler et al. [32], included a section asking healthcare professionals how they assessed pain. The healthcare professionals reported that they apply a wide range of pain assessment tools: e.g. behavioural observation, the Visual Analog Scale (VAS), the Visual Rating Scale (VRS), the Numeric Rating Scale (NRS), verbal assessment of pain, the Facial Pain Scale and Pain Assessment in Advanced Dementia (PAINAD).

The assessment of the drugs administered was achieved through retrospective chart review in all but one of the studies working with patient data. Morrison and Siu [12] was the only study to perform a daily chart review.

Nine studies stated that they had converted the opioids administered into morphine equivalents [10, 12, 21, 22, 29, 31–33, 35], while one study converted opioids as well as non-opioids administered into acetaminophen equivalents [34]. Four studies noted that they had adjusted their drug administration according the World Health Organization analgesic ladder [12, 23, 24, 26, 36]. The study by Holdgate et al. [27] simply documented all the drugs administered, whereas Hwang et al. [28] grouped the drugs administered into opioids and non-opioids. A more detailed look at this can be found in Table 4.

## Discussion

Fourteen of the included studies assume that pain management for PwD after hip fracture was insufficient. Interestingly, three of the five studies stating that the cognitive status did not play a significant role regarding administered pain medication were performed as studies at the same centres as previous studies by the same researchers: Feldt et al. [35] and Feldt & Gunderson [29] as subsequent studies to Feldt et al. [33]. There could be a bias due to halo effects from the previous studies, e.g. it can be assumed that the previous studies might have led to more sensitisation of the healthcare professionals in this setting. In the study by Feldt et al. [35] it was reported that nursing staff had received training relating to the findings of the previous study. One of the two hospitals included had even implemented extensive training for all nursing staff focusing on the Acute Pain Management Guidelines [35]. This may explain why the studies in the following years no longer found the differences that were observed previously [33, 35]. The study performed by Ardery et al. [21] is a different kind of a subsequent study: eight participants who had received no analgesia at all in the previous study by Titler et al. [32] were examined in more detail, but no new data was collected. The other two studies that did not find significant undertreatment of PwD after hip fracture but concluded that dementia is a major factor affecting drug-based pain management for this cohort had only a secondary focus on our research question [27, 28].

No studies were found that investigated pain management after pelvic fractures. Based on the findings regarding pain management after hip fracture, it may be assumed that pain management of pelvic fractures for PwD is a problem as well [4].

Considering the applied cognitive assessments, it can be assumed that these affected the results in a negative way, e.g. the assessment of mental status in the included studies was not optimal: Tombaugh et al. [37] stated that the Mini Mental State Exam (MMSE), which was applied in six of the studies, provides a brief screening of cognitive impairment and documents cognitive changes occurring over time, but it should not, by itself, be used as a diagnostic tool to identify dementia [37]. McDermott et al. [26], who made use of the AMTS, acknowledged in their publication that this tool is not diagnostic, but they argued that a number of studies had verified its validity and sensitivity for identifying PwD.

The three studies performed by Feldt et al. [29, 33, 35] and the study published by Grall [34] were the only studies that not only made use of patients’ self-reporting of pain but also applied behavioural and non-verbal tools to assess their patients’ pain. In the other studies it is not possible to know whether the pain of PwD was assessed correctly, due to the fact that these studies did not make use of standardised pain scales. Even though self-reporting of pain is the best approach in cognitively intact adults [38], it is not recommended for people with severe dementia because they are often unable to verbalise their pain or to compare it with the pain they experienced hours or days before [13]. The most suitable approach for pain assessment in this cohort is the use of observational scales [39]. Especially the PAINAD scale proved to be valid for PwD after hip fracture [38]. Even though the nurses interviewed by Rantala et al. [14] answered that they would use behavioural observation, facial pain scales and the PAINAD scale, this was not reported or observed in any of the other studies using chart reviews as a data assessment method. Another problem regarding pain assessment is that different pain scales might come to different results in the same patient cohort, as stated by Chibnall and Tait [40], who compared four different pain scales in cognitively impaired and unimpaired older adults. Takai et al. [41], who performed a literature review of pain prevalence among older adults, also came to the conclusion that the detection of the prevalence of pain appears to be related to research methods and data sources used as well as to the time frame.

Further, it is difficult to compare the different types of assessment of drug doses that were prescribed and/or administered to the patients: e.g. the study by Adunsky et al. [10] examined the opioids *administered* during the entire hospitalisation and converted them into morphine equivalents, while in the studies by Feldt et al. all the pain medication *prescribed and administered*, except acetaminophen, which was calculated separately, were converted into morphine equivalents [29, 33, 35]. The study by Grall [34], on the other hand, converted all the pain medication *prescribed* and *administered* during the 24 h following the first pain assessment and converted them into acetaminophen equivalents.

Interestingly, all of the eight studies that found undertreatment of drug-based pain management for people with dementia came to the conclusion that PwD received fewer opioids than people without dementia, while none of them found a significant difference in the non-opioids received [10, 12, 22, 26, 31–34]. A reason for this might be the fact that the caregivers are more afraid of the potential side effects of opioids in this vulnerable population, as stated by Feldt et al. [33]. The study by Mak et al. [31] stated that PwD received less analgesia during all the time periods, but no distinction was made between opioids and non-opioids. Based on the studies identified, it was not possible to conclude whether the undertreatment was due to lower doses or lower frequencies because it normally seems to be a combination of the two.

A review by Maidment et al. [42] stated that administration and prescription of medication are the two most common sources of error in older people with mental health problems: in the studies identified it seems to be an issue that in many cases the pain medication was prescribed PRN instead of being prescribed as a “standing order”. For example, in the studies performed by Ardery et al. [21] and Morrison and Siu [12] only a quarter of PwD received a standing order for an analgesic agent. This is not appropriate, since PwD do not ask for pain medication as frequently as other patient populations [21]. This should not be understood as resulting from decreased pain perception, but as being due to a number of different factors that affect the verbalisation of pain: e.g. fear of addiction or other adverse side effects, generational behaviours and attitudes, less-demanding behaviour, not wanting to appear a burden [21], and not being able to verbalise pain adequately due to cognitive impairment [12]. However, although the verbal approach to pain assessment was the most often used in the studies identified, it is not always the most appropriate. Effective PRN dosing for these patients would require the healthcare professionals to continually observe for signs of pain and provide medication prior to painful events, but most importantly the patients would have to be able to participate fully in the treatment plan [13] and to administer medication safely [43].

Another issue regarding pain medication administration could be the type of pain medication. For example, Feldt et al. [33] as well as Titler et al. [32] discussed that PwD should not be administered meripedine, due to the negative side effects of this drug in this vulnerable patient cohort. Considering the literature identified, it remains unclear whether different types of pain management may have impacts that are different on PwD than on people who are cognitively intact.

Finally, gender does not have a significant impact as a confounder, as analysed by Adunsky et al. [10] and Jensen-Dahm et al. [22].

Although the nursing home setting was pointed out as a relevant confounder by Feldt and Gunderson [29], none of the other included studies analysed this setting, and the three studies assessing the emergency department setting did not identify this as having a significant impact on the drug-based pain management in PwD after hip or pelvic fractures [26–28]. With all of this taken into account, we can only conclude that there is no difference to be found between the hospital and emergency department settings.

## Limitations

Since only two investigators performed selection of the studies there is the risk of selection bias. Due to the heterogeneity of the studies we were not able to perform a meta-analysis for all of the included studies.

Furthermore, three very promising abstracts [44–46] that seemed to focus on our research question to a great extent were not available, although we tried to contact the authors as well as the publishing journals.

## Conclusion

PwD do not seem to receive the same amount of opioid analgesics after hip fracture as people without cognitive impairment. Cognitive impairment seems to be a major barrier while assessing pain or administering drug-based pain medication in hip fracture patients. Healthcare professionals do not use the recommended scales for pain assessment of PwD, and they do not follow guidelines regarding the prescription of analgesics in this vulnerable patient cohort. One can conclude that the assessment of the patients’ mental status is essential before starting treatment.

There is a need to enhance pain assessment and management for PwD. Future research is needed to assess drug-based pain management for PwD after pelvic fractures, as well as more focused research regarding pain management for PwD after hip fractures, especially across settings (hospital to nursing home). Future studies should pay more attention to the use of appropriate items for the identification of PwD, and pain assessment in this patient cohort. The majority of the included studies were chart reviews with relatively small sample sizes in a hospital setting, even though observational and prospective studies with greater sample sizes in all settings would be much more suitable for this research question.

## Abbreviations

ED: Emergency department
NH: Nursing home
PRN: *pro re nata* (“as needed”) — medication that is administered only when asked for rather than on a regular basis/standing order
PwD: People with dementia
